# Three-port versus four-port laparoscopic cholecystectomy in acute and chronic cholecystitis

**DOI:** 10.1186/1471-2482-7-8

**Published:** 2007-06-13

**Authors:** Dhafir Al-Azawi, Nariman Houssein, Abu Bakir Rayis, Donal McMahon, Dermot J Hehir

**Affiliations:** 1 Department of surgery, Royal College of Surgeons in Ireland, Dublin Ireland; 2 Department of surgery, Tullamore regional Hospital, Tullamore, Co. Offally, Ireland; 3 School of Diagnostic imaging, University College Dublin, Dublin, Ireland; 4 School of Mathematical Sciences, University College Dublin, Dublin, Ireland

## Abstract

**Background:**

Several modifications have been introduced to laparoscopic cholecystectomy (LC). The three-port technique has been practiced on a limited scale. Our aim was to compare the three-port and four-port LC in acute (AC) and chronic cholecystitis (CC).

**Methods:**

The medical records of 495 patients who underwent LC between September 1999 and September 2003 were reviewed. Variables such as complications, operating time, conversion to open procedure, hospital stay, and analgesia requirements were compared.

**Results:**

Two hundred and eighty-three patients underwent three-port LC and 212 patients underwent four-port LC. In total, 163 (32.9%) patients were diagnosed with AC and 332 (67.1%) with CC by histology. There was no statistical difference between the three and four-port groups in terms of complications, conversion to open procedure (p = 0.6), and operating time (p = 0.4). Patients who underwent three-port LC required less opiate analgesia (pethidine) than those who underwent four-port LC (p = 0.0001). The hospital stay was found to be related to the amount of opiates consumed (p = 0.0001) and was significantly shorter in the three-port LC group (p = 0.005).

**Conclusion:**

Three-port LC is a safe procedure for AC and CC in expert hands. The procedure offers considerable advantages over the traditional four-port technique in the reduction of analgesia requirements and length of hospital stay.

## Background

Since its foundation in 1987 by Philip Mouret of Lyon, laparoscopic cholecystectomy (LC) has been the procedure of choice for symptomatic gall bladder disease [1]. Since then, there have been many changes and improvements in the technique. Traditional LC is performed using four-port technique [2,3]. Reducing the size or number of ports did not affect the safety of the procedure and further enhanced the advantages of laparoscopic over open cholecystectomy [4]. These modifications actually reduced the pain and analgesia requirement [5]. Three trocars and even two trocars were used to perform LC [4,6], as has using mini-instruments, authors of these new techniques claimed that these techniques took a similar time to perform and caused less postoperative pain than the standard laparoscopic cholecystectomy [5,7]. Some authors even advised for procedures as needlescope cholecystectomy to be practiced routinely [8]. The value of the lateral (fourth) trocar in the American technique used to hold the gall bladder fundus was challenged [9,10]. Recently published data [4,11] showed that three-port technique didn't compromise the procedure's safety. Reduction in analgesia requirement and cosmetic benefits were a common conclusion, however the procedure was performed on elective patients only in these published reports. In this large comparative study we compared the safety, outcome, and advantages between three-port and four-port LC in acute cholecystitis (AC) and chronic cholecystitis (CC).

## Methods

The medical records of 495 patients who underwent LC between September 1999 and September 2003 at Tullamore Regional Hospital in Ireland were reviewed retrospectively. Patients were identified by reviewing the operating theatre log books. A single consultant surgeon carried out the surgical procedures with an experience of more than 300 LC. After both procedures were explained in details, patients were given the option of choosing the operation to be performed by either three or four port laparoscopy techniques according to the advice given by the ethics committee and the training body given the fact that three-port LC is not the standard procedure for gall bladder stone disease. Written consent was taken. Elective patients were referred by general practitioners while emergency cases were admitted through the accident and emergency department. Preoperative work up include a complete history and physical examination, standard laboratory tests including liver function tests and radiological examinations including abdominal ultrasound. Ultrasonography confirmed the presence of gall bladder stones in all patients. Selective intraoperative cholangiogram (IOC) policy was used, being performed only for those cases in which choledocholithiasis were suspected on clinical, laboratory ground and in cases where anatomy appeared unclear at operation.

### Laparoscopic cholecystectomy techniques

The three-port technique involves inserting a 10 mm trocar (Bladeless trochar – Johnson &Johnson) just above the umbilicus using the open technique (Hasson's technique) through which the zero viewing videoscope (Olympus) was introduced. Another 10 mm trocar (Endopath Tristar trocar – Johnson & Johnson) was inserted 3 cm below the xiphesternum; and finally, a 5 mm trocar (Endopath Tristar trocar) at the right hypochondrium anterior axillary line 3 cm below the costal margin. The operating surgeon conducted the procedure from the left side of the patient together with the assistant holding the camera while the TV monitor was located on the upper left side of the patient and the nurse on the lower left side of the patient. The operating surgeon holds the dissecting instruments with his right hand through the 10 mm trocar while holding the gall bladder at the infundibulum with a grasper through the 5 mm trocar, moving the infundibulum right and left or back and forth to display Calot's triangle, blunt dissection was used for adequate display of the cystic duct and cystic artery. The cystic duct was then clipped and divided followed by the cystic artery. The gall bladder was then dissected from its bed and extracted from either the umbilical or the subxephesternal ports. IOC was performed through the 10 mm subxephesternal trocar.

The four-port LC was performed using the North American 'flip over' technique [12].

### Post-operative analgesia requirement

After surgery patients were taken to the post-anaesthesia care unit after which they were taken to the inpatient ward where they were given analgesics (pethidine and or diclofenac) unless allergies or specific contraindications were noted. Patients received their analgesics according to their pain ratings measured by nursing staff using the verbal rating scale [13]. The total amount of analgesia required by each patient was calculated over 48 hours after surgery. Discharge from hospital was made as a joint decision between nursing staff, the surgical team and patients using an early discharge planning rating scale applied by the department of surgery in the hospital [14].

### Statistical tests

Continuous variables were calculated as mean and compared using the two-tailed Student's t test p value of less than 0.05 considered significant. Ordinal variables were calculated as median (range) and compared using the Mann-Whitney U test. This test was used to analyse differences in operating time and hospital stay between the two groups. When modelled separately, the test was chosen to block by a factor outlining whether each patient's condition was acute or chronic. This allowed for expected differences across the two forms of cholecystitis; hence, the models isolated (as much as was possible) the differences in behaviour due solely to the surgery type. Similarly, it was chosen to block by patient age, but this was found not to be necessary and was thus excluded from the analysis. Bivariate correlations were used to measure relations between variables (amount of pethidine and hospital stay). Statistical evaluations were performed using SPSS version 11.0 (SPSS, Chicago, Illinois, USA). While Logistic modelling was carried out on the related data (conversion rate and complications) p value of less than 0.05 considered significant using Proc Genmod in SAS version 8.2 statistical program (SAS Institute, Cary, North Carolina, USA).

## Results

Four hundred and ninety-five patients underwent laparoscopic cholecystectomy; 399 were female and 96 were male. The age range was 16–83 years, with a mean age of 50 years. The three-port LC technique was performed on 283 (57.2%) patients, while the traditional four-port LC technique was performed on 212 (42.8%) patients. One hundred and sixty-three (32.9%) patients were diagnosed with AC and 332 (67.1%) patients were diagnosed with CC by histology, table 1. IOC was performed on 38 patients in the three-port group and 32 patients in the four-port group. None of the three-port LC group needed a fourth port to complete the procedure.

**Table 1:** patients distribution according to the gall bladder histology and the type of LC procedure performed.

		**Number of Ports**		
			
		**Three-port**	**Four-port**	**Total**
**Gall Bladder Histology**	**Acute Cholecystitis**	97 (34.3%)	66 (31.1%)	163 (32.9%)
	**Chronic Cholecystitis**	186 (65.7%)	146 (68.9%)	332 (67.1%)

**Total**		283 (57.2%)	212 (42.8%)	495 (100%)

### Postoperative analgesia requirement

**Figure 1:**
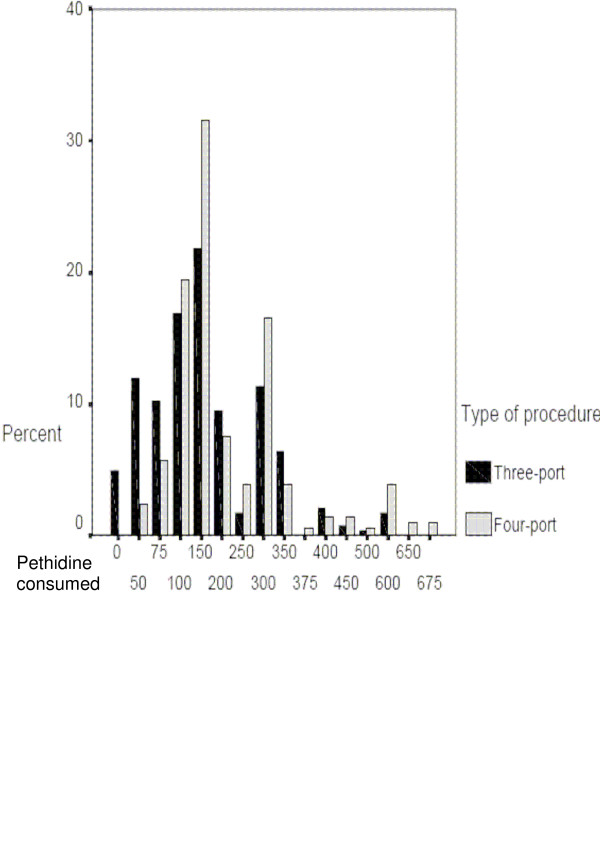
pethidine requirement. The Bar chart shows the percent of patients received pethidine (mg) in the first 48 hours after surgery. The black bars represent three-port LC patients while the grey bars represent four-port group, means are 167.23 mg and 210.73 mg respectively *p *= 0.0001.

The average (mean) amount of pethidine consumed by each patient during the first 48 hours after three-port and four-port LC were 167.23 mg and 210.73 mg respectively. The amount of diclofenac consumed for the same period was found to only relate to whether the patient was acute or chronic (p = 0.001). The amount of pethidine, however, was found to have a significant relationship to whether three- or four-port surgery was performed p = 0.0001, figure 1. The average verbal pain scale of three port LC patients was found to be significantly lower than four port LC patients p = 0.003, table 2.

**Table 2:** The difference in verbal pain scale between the two groups.

	**Low pain Scale**	**High pain Scale**	**Total**
**Three Port**	230 (60.7%)	53 (45.7%)	283
**Four Port**	149 (39.3%)	63 (54.3%)	212
**Total**	379	116	

The verbal pain scale is a scale of four grades; the average pain scale score was calculated for each patient. Grades 1 and 2 were grouped as low pain scale while grades 3 and 4 were graded as high pain scale.

### Operating time and length of hospital stay

**Figure 2:**
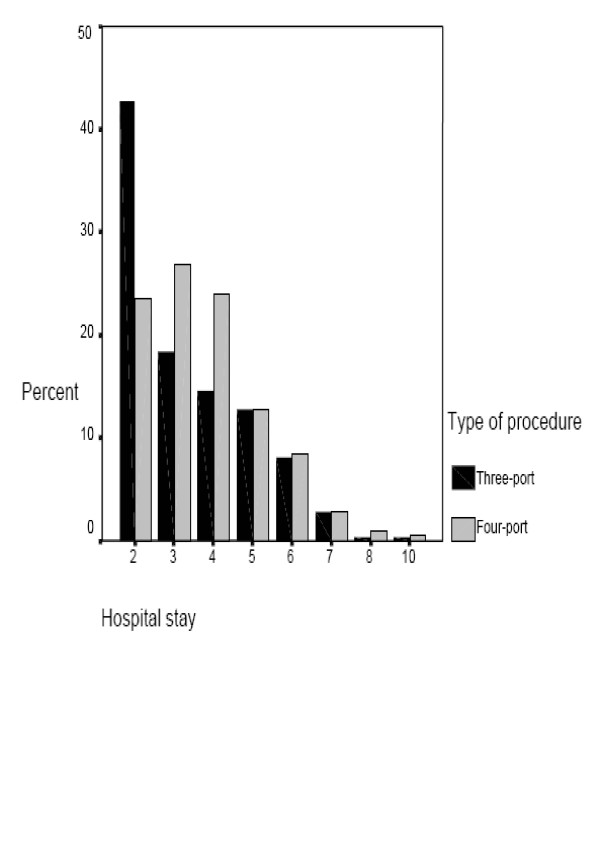
Hospital stay. The bar chart demonstrates the differences in hospital stay (days) between the three-port LC group (black bars) and the four-port LC group (grey bars). Three-port LC patients stayed less in hospital in comparison to the other group *p *= 0.005.

**Figure 3:**
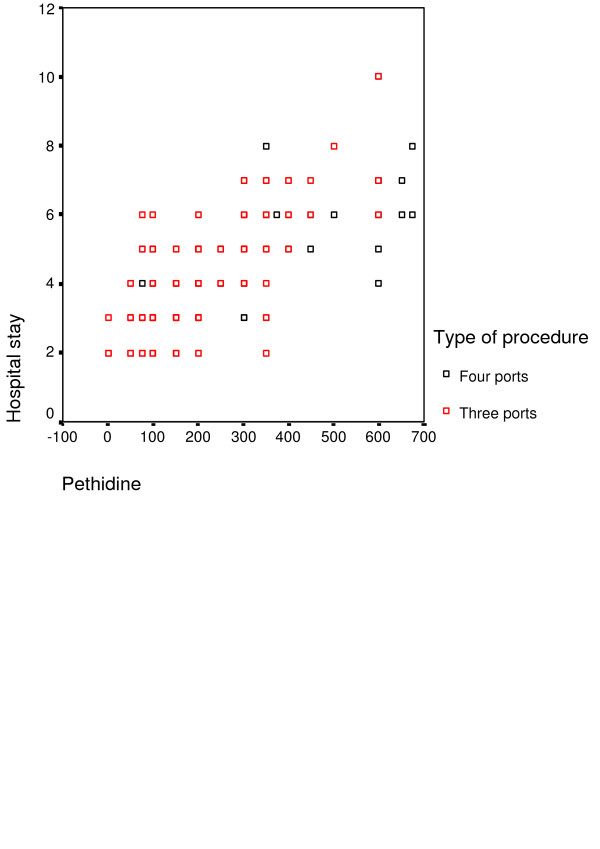
the correlation between the hospital stay and the amount of pethidine required. The scatter plot illustrates the linear correlation between hospital stay and the amount of pethidine consumed by patients (red plots resemble three-port group and the black plots represent four-port group).

The mean operating time for the three-port LC procedure was 46.1 minutes versus 48.9 minutes for the four-port technique. No significant difference between the two techniques (p = 0.4). However, when length of hospital stay was examined, there was a significant decrease of hospital stay in the three-port technique compared to the traditional LC: mean hospital stay was 2.8 days and 3.7 days, respectively p = 0.005, figure 2. Bivariate correlation showed that the length of hospital stay was significantly related to the amount of pethidine consumed by patients in both groups p = 0.0001, figure 3.

### Postoperative complications and conversion rate

There were no common bile duct injuries or deaths reported in both groups. Conversion to open procedure and post operative complications (port site bleeding, wound infection, wound haematoma, pleural effusion, abdominal pain, and jaundice) outlined in table 3. There were no significant differences between the two types of procedures in terms of postoperative complications as well as between acute and chronic cholecystitis patients, table 4.

**Table 3:** The percent of conversion rate and complications among the two groups. No significant differences were noticed.

	Three-Port	Four-Port	*p*-Value
Conversion to open procedure	8 (2.8%)	6 (2.8%)	0.6
Port site bleeding	1 (0.4%)	4 (1.9%)	0.1
Wound infection	4 (1.9%)	4 (1.9%)	0.47
Wound haematoma	0	3 (1.4%)	0.078
Pleural effusion	2 (0.7%)	4 (1.9%)	0.21
Abdominal pain	8 (2.8%)	8 (3.8%)	0.36
Jaundice	1 (0.4%)	0	0.57

**Table 4:** The differences in outcome between acute and chronic cholecystitis patients.

	**Acute Cholecystitis**	**Chronic cholecystitis**	**P Value**
**Conversion to open**	7	7	0.138
**Port site bleeding**	3	2	0.203
**Abdominal pain**	7	8	0.248
**Wound infection**	3	5	0.523
**Wound haematoma**	0	3	0.301
**Pleural effusion**	4	2	0.095

## Discussion

There have been a number of modifications in the technique of LC. The use of the fourth trocar which is generally used for fundic retraction in the American technique seemed unnecessary by some surgeons [4] others used sutures to retract the gall bladder [11,15]. Trichac in his prospective trial addressed the safety and the advantages of the three port technique in terms of analgesia requirement [11], though he found no improvement in postoperative hospital stay, his work and other published series on this technique were carried out only on elective patients. In fact the procedure was practiced on cases of acute cholecystitis as well but not reported [16]. In this retrospective single centre non randomised review we compared the safety and the advantages of three-port LC in AC and CC in a large comparative study.

When performed on acute and chronic cholecystitis the three-port technique was found to be safe; there were no common bile duct injuries or deaths in either group. Port site bleeding, haematoma at the port sites, and pleural effusion were encountered less frequently in patients who underwent three-port LC however the differences were not statistically significant.

The three-port technique did not change the rate of conversion when compared to the four-port technique or to data published in the literature [17,18] also the operating time did not increase as a result of this technique when performed in both types of cholecystitis, even when compared to published series [19,20].

Diclofenac and pethidine were the most common postoperative analgesics prescribed after LC [21]. Vomiting and excessive sedation are known side effects of pethidine. Patients who underwent three-port procedure needed less pethidine than those who underwent four-port LC. On the other hand diclofenac intake did not relate to the number of ports used.

Although the length of hospital stay is longer than expected for both groups due to the fact that patients were admitted one day prior to surgery at the time of conducting the study. The introduction of the three-port technique improved the length of hospital stay, adding another cost-effective benefit to the procedure; looking for possible causes for this reduction we found a strong correlation between the amount of opiates consumed and the length of hospital stay which may in part explain this reduction.

In conclusion, in spite of the limitations of our study being a retrospective review, we found that the use of three ports in LC did not affect the procedure's safety, conversion rate, and operating time when used in AC and CC. The introduction of the three-port technique, which is still in routine practice in our institute, meant patients, needed fewer painkillers and shorter hospital stays; other advantages include fewer scars and more cost savings. However this technique has its own limitations; at present we recommend it to be only practiced by surgeons experienced in laparoscopic techniques.

## Competing interests

The author(s) declare that they have no competing interests.

## Authors' contributions

DA designed the study, collected the data and wrote the manuscript, NH managed, coordinated the design and critically reviewed the manuscript, DM performed the statistical analysis, BR contributed in the design of the study and DH conceived the study. All authors read and approved the manuscript.

## Pre-publication history

The pre-publication history for this paper can be accessed here:


